# The clinical value of the neutrophil-to-lymphocyte ratio, systemic immune-inflammation index, monocyte-to-lymphocyte ratio and platelet-to-lymphocyte ratio for predicting the severity of patients with autoimmune encephalitis

**DOI:** 10.3389/fneur.2025.1498007

**Published:** 2025-02-28

**Authors:** Xin Zhao, Fen Wu, Shunfeng Zhao, Wenna Chen, Wei Si, Yuanrui Li, Dengke Zhang, Jing Wang, Ningning Wang, Lina Sun, Zhiyu Sun, Haoxiao Chang, Ganqin Du

**Affiliations:** ^1^Department of Neurology, The First Affiliated Hospital, College of Clinical Medicine of Henan University of Science and Technology, Luoyang, China; ^2^Department of Clinical Laboratory, Liaocheng Third People’s Hospital, Liaocheng, China; ^3^China National Clinical Research Center for Neurological Diseases, Beijing Tiantan Hospital, Capital Medical University, Beijing, China; ^4^Department of Neurology, Tianjin Medical University General Hospital, Tianjin, China; ^5^Department of Neurobiology, School of Basic Medical Sciences, Capital Medical University, Beijing, China

**Keywords:** autoimmune encephalitis, systemic inflammation index, neutrophil-to-lymphocyte ratio, monocyte-to-lymphocyte ratio, platelet-to-lymphocyte ratio, severity, intensive care unit

## Abstract

**Background:**

The systemic inflammation index (SII), neutrophil-to-lymphocyte ratio (NLR), monocyte-to-lymphocyte ratio (MLR) and platelet-to-lymphocyte ratio (PLR) are inflammatory markers in peripheral blood, which have been proven to be associated with some central nervous system diseases. We aimed to evaluate the association of SII, NLR MLR and PLR with the severity of autoimmune encephalitis (AE) and to compare the predictive value of those biomarkers in the early identification of ICU admission.

**Methods:**

This retrospective study was conducted in three medical centers in China. We included 176 patients diagnosed with AE and 200 age and gender-matched healthy controls and correlated their demographic and clinical data. The SII, NLR, MLR and PLR levels were calculated from the blood routine tests. The severity of the patients was evaluated by the Clinical Assessment Scale for Autoimmune Encephalitis (CASE) and the modified Rankin Scale (mRS) at admission, and the patients were divided into two groups according to the ICU admission.

**Results:**

The SII, NLR, MLR and PLR were significantly higher in AE patients than that in HCs (<0.001 for all). The SII and NLR were positively correlated with the CASE score (*r* = 0.243, *p* = 0.001; *r* = 0.237, *p* = 0.002) and the mRS score (*r* = 0.185, *p* = 0.014; *r* = 0.185, *p* = 0.014) in AE patients. The MLR and PLR were only positively correlated with the CASE score (*r* = 0.242, *p* = 0.001; *r* = 0.158, *p* = 0.036). The SII and NLR of the ICU group were significantly higher than that of the non-ICU group. The result of receiver operating characteristic (ROC) analysis showed that NLR was the best predictor of ICU admission for AE patients (AUC = 0.701). NLR and MLR had similar predictive ability (AUC = 0.654; AUC = 0.608) and were superior to PLR. The optimal NLR cut-off value for the incidence of ICU was 3.906.

**Conclusion:**

Increased SII, NLR, MLR and PLR at admission are positively correlated with the CASE score of AE patients. Among the four indexes, the NLR is the best predictor of ICU admission, which may be helpful for clinicians to monitor disease progression and identify potentially severe patients of AE.

## 1 Introduction

Autoimmune encephalitis (AE) constitutes a group of inflammatory diseases of the central nervous system characterized by immune-mediated inflammation of the brain and disruption of neural circuits (1). The concept of AE was proposed in 2007 following the discovery of anti-N-methyl-D-aspartate receptor (NMDAR) antibodies in encephalitis patients (2). Subsequently, various subtypes of AE were recognized, including leucine-rich glioma inactivated 1 (LGI1) encephalitis, gaminobutyric acid type B receptor (GABABR) encephalitis, and other types of encephalitis (3). The pathogenesis of AE is not fully understood, but research suggests that certain infections, medications, or underlying cancers may trigger AE. The manifestations and prognosis of AE have significant clinical heterogeneity. Some patients experience rapid progression, which can be life-threatening due to central hypoventilation or severe autonomic nervous dysfunction within weeks or even days. This underscores the importance of monitoring disease progression as a clinical priority in AE management (1). Although the related antibodies are important biomarkers for AE, their titers correlate imperfectly with the course of the disease, meanwhile, the process of antibody testing is often prolonged and not consistently accessible (4). Currently, there are no definitive biological indicators that have been confirmed to be precise and effective in predicting the prognosis of AE (3).

The systemic inflammation index (SII), neutrophil-to-lymphocyte ratio (NLR), monocyte-to-lymphocyte ratio (MLR) and platelet-to-lymphocyte ratio (PLR) are markers derived from blood routine, showed sensitivity in indicating the inflammatory reaction. Previous studies have found a strong correlation between those blood inflammatory markers and systemic diseases such as tumors and autoimmune diseases (5–8). Recently, some studies found that those biomarkers can reflect the inflammatory activity of central nervous system diseases. A systematic review has indicated a correlation between the SII and the severity and prognosis of acute stroke (9). Another study showed that MLR was positively related to the severity and activity of multiple sclerosis and some authors hold that increased PLR was related to the incidence and poor prognosis of post-stroke depression (10, 11).

As far as we know, few studies have focused on the predictive value of these inflammatory markers for the severity of AE. Moreover, no studies have compared these inflammatory markers in AE patients. This study systematically explored differences in SII, NLR, MLR, and PLR between AE patients and healthy individuals. We also explored in depth their relevance to Assessment Scale in Autoimmune Encephalitis (CASE) and modified Rankin Scale (mRS) scores and compared the predictive abilities of these biomarkers for predicting intensive care unit (ICU) incidence in AE patients to find a reliable and cost-effective biomarker to evaluate the severity of AE.

## 2 Subjects and methods

### 2.1 Patients

A total of 176 patients diagnosed with AE and 200 age and sex-matched healthy individuals as control were recruited in the First Affiliated Hospital of Henan University of Science and Technology, the Third People’s Hospital of Liao Cheng and Beijing Tiantan Hospital from January 2019 to October 2023. This study was conducted following the ethical principles of the Declaration of Helsinki and was approved by the Human Ethics Review Board in all participating medical centers. The inclusion criteria included: (1) over 18 years old; (2) meeting the diagnostic criteria for AE (1); (3) positive AE-related antibodies in serum and/or cerebrospinal fluid (CSF) tests; (4) complete clinical data; (5) consent to participate in the study. The exclusion criteria included: (1) complicated with other acute neurological diseases; (2) previous dyskinesia; (3) complicated with other systemic autoimmune diseases, such as systemic lupus erythematosus; (4) comorbidity may affect blood routine, such as infectious, neoplastic and hematological diseases; (5) have received immunotherapies, including corticosteroids, steroid-sparing agents, intravenous immunoglobulin (IVIG), plasma exchange, rituximab, cyclophosphamide, or other treatments potentially affecting peripheral immune cells, within 4 weeks before admission.

### 2.2 Data collection

The detailed information, such as age of onset, gender, clinical manifestations, mRS scores, CASE scores, blood routine tests and the percentage of ICU admissions, was collected from the electronic medical record system. The mRS scores, CASE scores and the percentage of ICU admissions were pre-specified as important clinical outcomes based on their relevance to disease severity and patient management. The blood routine tests used to calculate the SII, NLR, MLR and PLR were all performed within 24 h after admission and before immunotherapy. SII = platelet × neutrophil/lymphocyte, NLR = neutrophil/lymphocyte, MLR = monocyte/lymphocyte, PLR = platelet/lymphocyte. The blood routine tests were conducted using a Beckman Coulter automated hematology analyzer. Antibody testing was performed in both serum and CSF using indirect immunofluorescence assays at the Guangzhou KingMed Center for Clinical Laboratory. Disease severity of AE was assessed at admission by the mRS scores and CASE scores, respectively. The mRS scale ranges from 0 (no symptoms) to 6 (death), with intermediate levels assessing various degrees of disability. The total scores of the mRS are 6 points. The CASE scale is divided into nine items, including seizures, memory dysfunction, psychiatric symptoms, consciousness, language problems, dyskinesia, gait instability and ataxia, brainstem dysfunction, and weakness. The total scores of the CASE are 27 points. All patients were divided into ICU and non-ICU groups based on admission. ICU admission was necessary if the patient had one or more of the following conditions: status epilepticus, delirium, coma, respiratory failure, severe sepsis, organ failure, need for mechanical ventilation or vasopressor support, a specific APACHE II score, or other complications such as resuscitation and increased intracranial pressure. Patients who remained in the ICU for at least 48 h were included in the ICU group.

### 2.3 Statistical analysis

Normally distributed continuous variables were presented as mean ± standard deviation (SD) and compared using an independent samples t-test. Non-normally distributed continuous variables were expressed as median and interquartile range (M, IQR) and analyzed using the Mann–Whitney U test. Categorical variables, reported as counts or percentages, were compared using the chi-square test. Spearman correlation was used to assess the relationship between SII, NLR, MLR, and PLR with disease severity. Receiver operating characteristic (ROC) curve analysis and the corresponding area under the curve (AUC) were used to evaluate the predictive value of SII, NLR, MLR, and PLR for ICU admission. A *p*-value <0.05 was considered statistically significant. Statistical analyses were conducted using IBM SPSS Statistics for Windows (version 22), and figures were generated using GraphPad Prism 9.5.

## 3 Results

### 3.1 Baseline characteristics of patients

**Figure 1:**
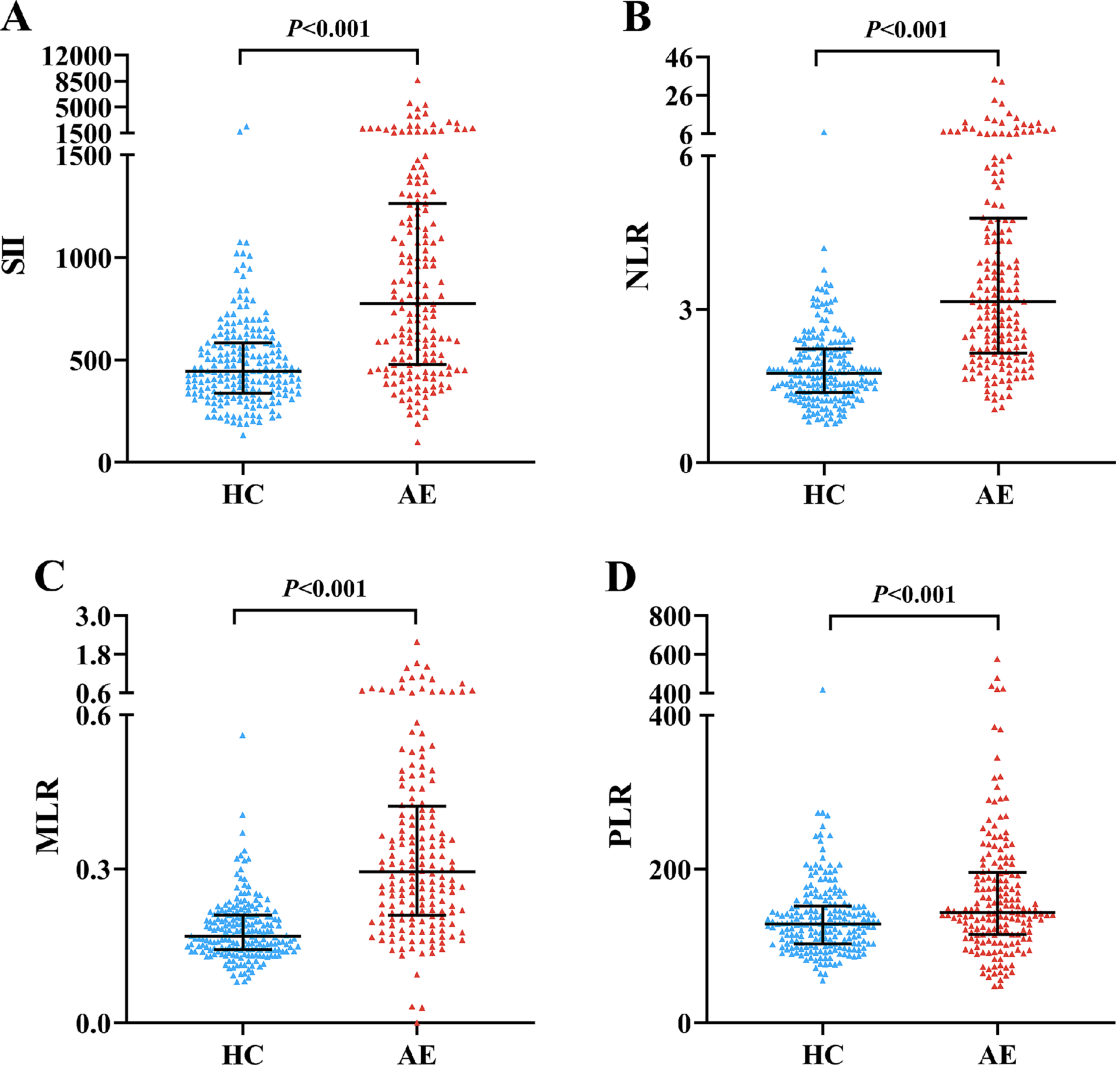
Comparison of SII, NLR, MLR and PLR between AE patients and HCs. Boxplots of the SII, NLR, MLR and PLR showing the distribution in the AE group and HC group. The SII of the AE group was higher than that of the HC group **(A)**. The NLR of the AE group was higher than that of the HC group **(B)**. The MLR of the AE group was higher than that of the HC group **(C)**. The PLR of the AE group was higher than that of the HC group **(D)**. AE, autoimmune encephalitis; HC, healthy control; SII, systemic immune-inflammation index; NLR, neutrophil-to-lymphocyte ratio; MLR, monocyte-to-lymphocyte ratio; PLR, platelet-to-lymphocyte ratio.

We enrolled 176 patients with AE and 200 age and gender-matched healthy controls. Among the 176 AE patients, there were 89 NMDAR encephalitis (50.57%), 47 LGI1 encephalitis (26.7%), 10 GABABR encephalitis (5.68%), 7 CASPR2 encephalitis (3.98%) and 23 other encephalitis (13.07%). The CASE score and mRS score at admission were 7 (4–13) and 2 (1–3), respectively. Table 1 shows the clinical and laboratory parameters of AE patients and HCs of our population. AE patients had higher WBC, neutrophils and monocytes than the HC group (*p* < 0.001). On the contrary, HCs had higher lymphocytes and platelets (*p* < 0.001) (Table 1). Compared with HCs, AE patients had significantly higher SII (774.34 vs. 444.82, *p* < 0.001), NLR (3.14 vs. 1.75, *p* < 0.001), MLR (0.3 vs. 0.17, *p* < 0.001), and PLR (142.86 vs. 128.62, *p* < 0.001) (Figure 1).

**Table 1:** Demographics and clinical data of the AE patients and HCs.

	AE (*n* = 176)	HC (*n* = 200)	*p* value
Sex, No (%)			0.492^a^
Male	95 (54.0%)	115 (57.5%)	
Female	81 (46.0%)	85 (42.5%)	
Age (year, Mean ± SD)	42.02 ± 18.83	40.02 ± 9.94	0.207^b^
WBC, 10^9^/L	8.13 (6.19–10.70)	6.17 (5.19–7.11)	<0.001^c^
Neutrophils, 10^9^/L (M, IQR)	5.38 (3.95–7.86)	3.50 (2.78–4.32)	<0.001^c^
Lymphocytes, 10^9^/L (M, IQR)	1.65 (1.27–2.08)	2.07 (1.71–2.49)	<0.001^c^
Monocytes, 10^9^/L (M, IQR)	0.50 (0.35–0.68)	0.36 (0.27–0.44)	<0.001^c^
Platelets, 10^9^/L (M, IQR)	231.00 (196.00–278.00)	253.50 (225.25–302.50)	0.001^c^
SII (M, IQR)	774.34 (477.16–1264.37)	444.82 (337.58–584.25)	<0.001^c^
NLR (M, IQR)	3.14 (2.14–4.79)	1.75 (1.37–2.23)	<0.001^c^
MLR (M, IQR)	0.30 (0.21–0.42)	0.17 (0.14–0.21)	<0.001^c^
PLR (M, IQR)	142.86 (114.79–194.92)	128.62 (102.80–151.95)	<0.001^c^
CASE (M, IQR)	7 (4–13)		
mRS (M, IQR)	2 (1–3)		
Antibody (%)			
NMDAR	89 (50.57%)		
LGI1	47 (26.70%)		
GABA	10 (5.68%)		
CASPR2	7 (3.98%)		
AMPAR	2 (1.14%)		
GAD65	8 (4.55%)		
DPPX	1 (0.57%)		
mGluR1	1 (0.57%)		
mGluR5	1 (0.57%)		
lgLON5	2 (1.14%)		
GlyR1	1 (0.57%)		
Amphiphy	2 (1.14%)		
CV2	1 (0.57%)		
Yo	1 (0.57%)		
GAD65, Hu	1 (0.57%)		
AMPAR, AMPH, CV2	1 (0.57%)		
NMDA, GAD65	1 (0.57%)		

HC, Healthy control; AE, Autoimmune encephalitis; M, Median; IQR, Interquartile range; WBC, White blood cell; NLR, Neutrophil-to-lymphocyte ratio; MLR, Monocyte-to-lymphocyte ratio; PLR, Platelet-to-lymphocyte ratio; SII, Systemic immune-inflammation index; CASE, The clinical assessment scale for autoimmune encephalitis; mRS, Modified rankin scale; NMDAR, Anti-N-methyl-D-aspartate receptor; LGI-1, Leucine-rich glioma inactivated 1; GABA, G-aminobutyric acid type; CASPR2, Contactin associated protein 2; AMPAR, Amino-3-hydroxy-5-hydroxy-5-methyl-4-isoxazolepropionic acid receptor; GAD65, Glutamic acid decarboxylase-65; DPPX, Dipeptidyl-peptiddase-like protein-6; mGluR1, Metabotropic Glutamate Receptor 1; mGluR5, Metabotropic Glutamate Receptor 5; GlyR1, Glycine receptor-1.

a Pearson’s chi-square test.

b The independent samples T test.

c Mann-Whitney U test.

### 3.2 Correlations of SII, NLR, MLR and PLR with the severity of AE

**Figure 2:**
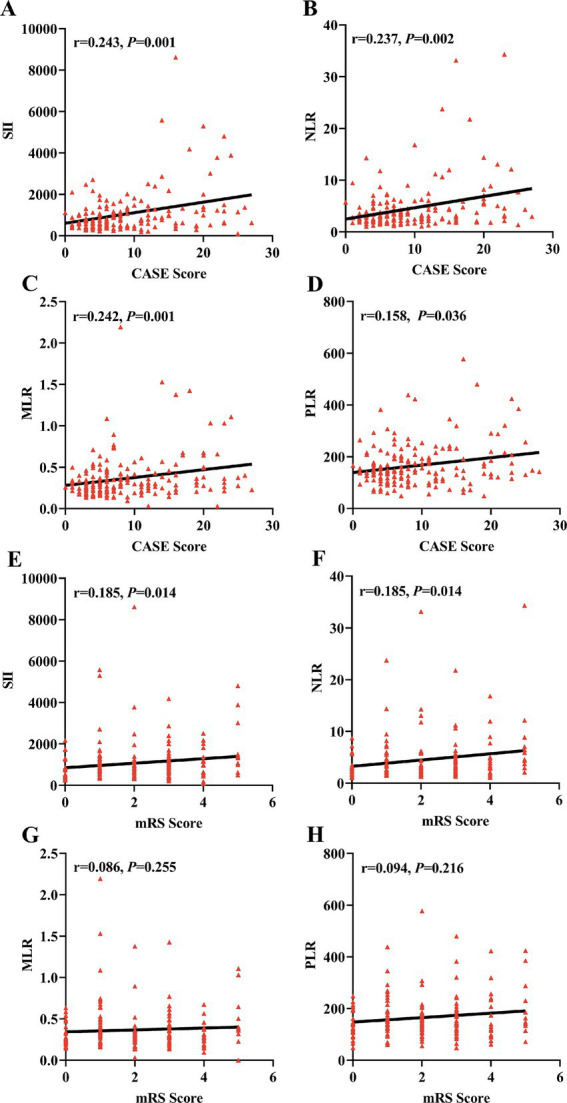
Correlations of SII, NLR, MLR and PLR with the severity of AE. The correlations of SII, NLR, MLR, and PLR with CASE score and mRS score were assessed using Spearman correlation analysis. The SII, NLR, MLR, and PLR were positively correlated with the CASE score in the AE group **(A–D)**. The SII and NLR were positively correlated with the mRS score in the AE group **(E,F)**. No correlation was observed between the MLR and PLR and the mRS score **(G,H)**. AE, autoimmune encephalitis; CASE, Clinical Assessment Scale in Autoimmune Encephalitis; mRS, modified Rankin Scale; SII, systemic immune-inflammation index; NLR, neutrophil-to-lymphocyte ratio; MLR, monocyte-to-lymphocyte ratio; PLR, platelet-to-lymphocyte ratio.

As shown in Figure 2, Spearman correlation analysis showed that SII and NLR were positively correlated with the CASE score (*r* = 0.243, *p* = 0.001; *r* = 0.237, *p* = 0.002) and the mRS score (*r* = 0.185, *p* = 0.014; *r* = 0.185, *p* = 0.014) in AE patients. Interestingly, we also found that MLR and PLR were positively correlated with the CASE score (*r* = 0.242, *p* = 0.001; *r* = 0.158, *p* = 0.036), but no correlation was observed between the mRS score and MLR or PLR (*r* = 0.086, *p* < 0.255; *r* = 0.094, *p* < 0.216).

### 3.3 The predictive value of SII, NLR, MLR and PLR on the ICU admission

**Figure 3:**
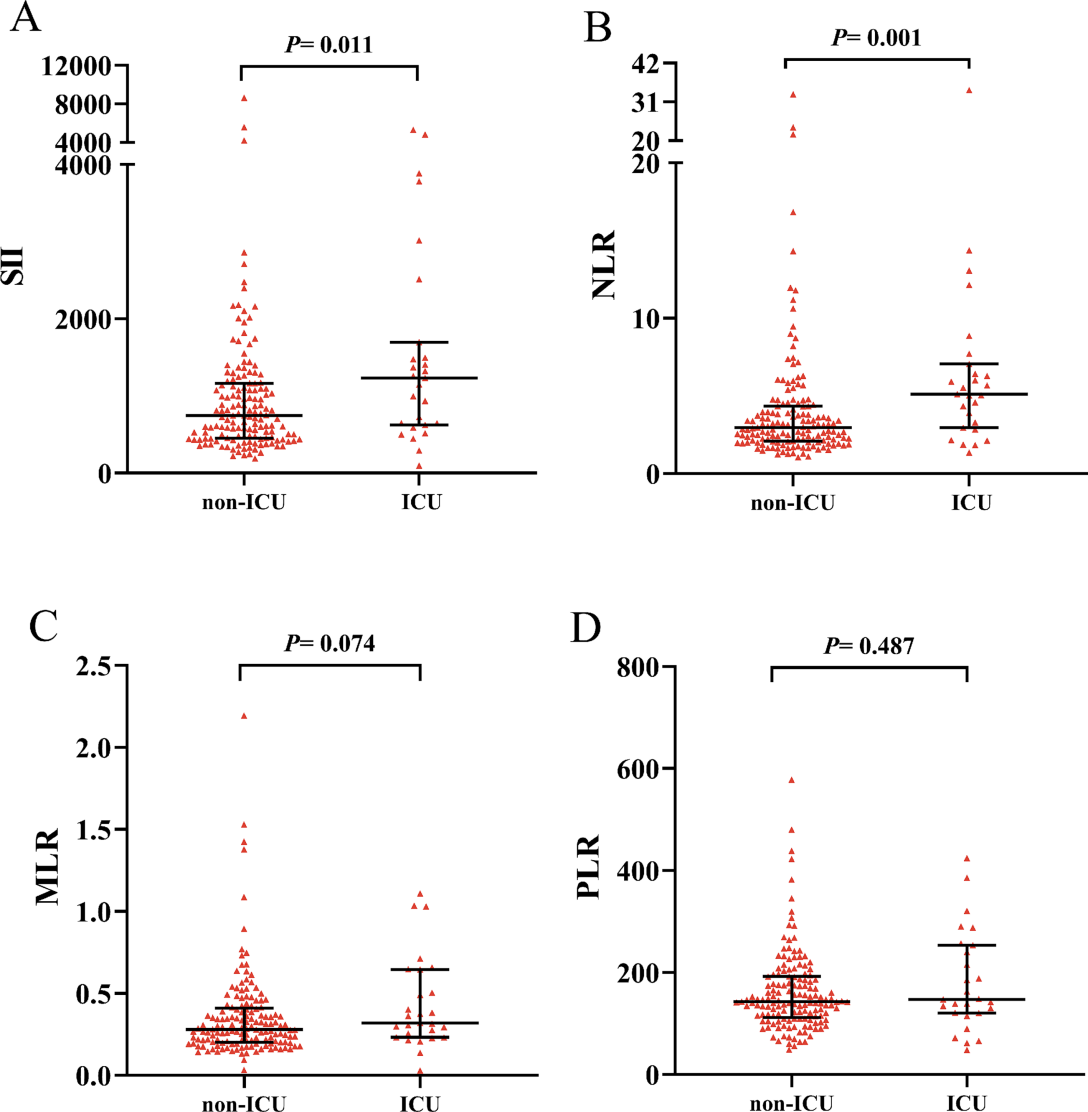
Comparison of SII, NLR, MLR and PLR between ICU group and non-ICU group. Boxplots of the SII, NLR, MLR and PLR showing the distribution in the ICU group and non-ICU group. The SII and NLR of the ICU group were higher than those of the non-ICU group **(A,B)**. The MLR and PLR were similar in two groups **(C,D)**. ICU, intensive care unit; SII, systemic immune-inflammation index; NLR, neutrophil-to-lymphocyte ratio; MLR, monocyte-to-lymphocyte ratio; PLR, platelet-to-lymphocyte ratio.

**Figure 4:**
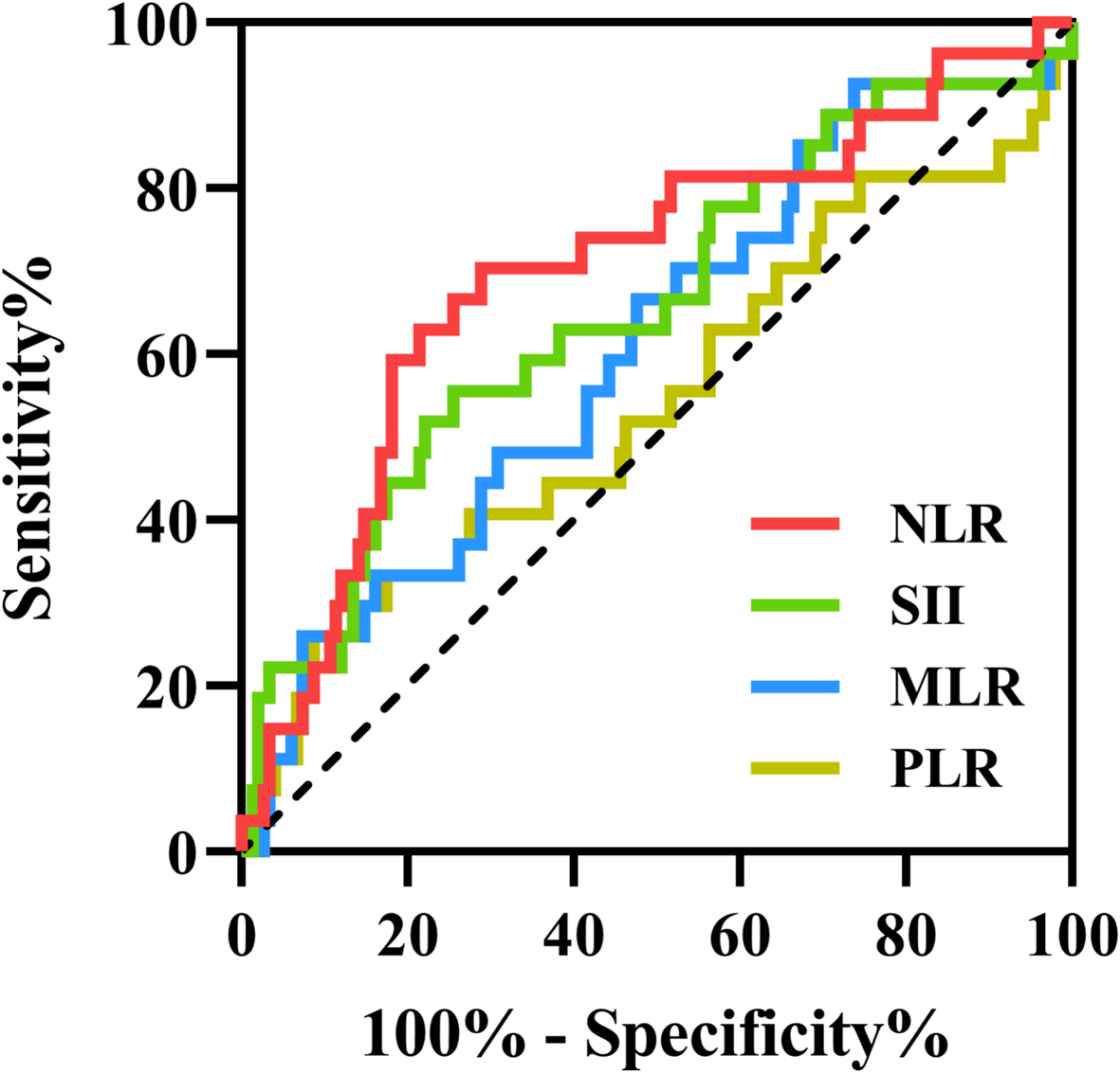
The predictive value of SII, NLR, MLR and PLR on the ICU admission. ROC curves of the SII, NLR, MLR and PLR for predicting ICU admission in AE patients. ROC, receiver operating characteristic; ICU, intensive care unit; AE, autoimmune encephalitis; SII, systemic immune-inflammation index; NLR, neutrophil-to-lymphocyte ratio; MLR, monocyte-to-lymphocyte ratio; PLR, platelet-to-lymphocyte ratio.

A total of 27 patients (15.3%) were admitted to the ICU, with significantly higher CASE sore (20 vs. 6, *p* < 0.001) and mRS score (4 vs. 1, *p* < 0.001) (Table 2). AE Patients in the ICU group showed significantly higher SII (1232.84 vs. 739.39, *p* = 0.011) and NLR (5.11 vs. 2.94, *p* < 0.001) than the non-ICU group. However, MLR and PLR were similar in two groups (0.32 vs. 0.28, *p* = 0.074; 147.66 vs. 142.78, *p* = 0.487) (Figure 3). ROC curve was conducted to analyze the ability of NLR, SII, MLR and PLR to predict the ICU admission in AE patients, respectively (Figure 4). Details of the optimal cutoff, specificity and sensitivity rates are shown in Table 3. Based on the AUC values, the NLR (AUC: 0.701, 95% CI: 0.588–0.815) showed the best predictive ability for ICU admission in AE patients, then followed by SII (AUC: 0.654, 95% CI: 0.535–0.774) and MLR (AUC: 0.608, 95% CI: 0.492–0.725). The PLR was the weakest predictor for ICU admission in AE patients (AUC: 0.542, 95% CI: 0.411–0.673). The optimal NLR cut-off value for the incidence of ICU was 3.906.

**Table 2:** Comparison of clinical data between non-ICU group and ICU group.

	non-ICU (*n* = 149)	ICU (*n* = 27)	*p* value
Sex, No (%)			0.858^a^
Male	80 (53.69%)	15 (55.56%)	
Female	69 (46.31%)	12 (44.44%)	
Age (year, Mean ± SD)	43.05 ± 18.99	36.30 ± 17.16	0.086^b^
SII (M, IQR)	739.39 (452.01–1164.45)	1232.84 (626.04–1697.41)	0.011^c^
NLR (M, IQR)	2.94 (2.08–4.35)	5.11 (2.95–7.07)	0.001^c^
MLR (M, IQR)	0.28 (0.20–0.41)	0.32 (0.23–0.65)	0.074^c^
PLR (M, IQR)	142.78 (111.32–191.87)	147.66 (121.03–253.61)	0.487^c^
mRS (M, IQR)	2 (1–3)	4 (3–5)	<0.001^c^
CASE (M, IQR)	6 (4–11)	20 (12–23)	<0.001^c^

SII, Systemic immune-inflammation index; NLR, Neutrophil-to-lymphocyte ratio; MLR, Monocyte-to-lymphocyte ratio; PLR, Platelet-to-lymphocyte ratio; mRS, Modified Rankin Scale; CASE, Clinical Assessment Scale in Autoimmune Encephalitis; non-ICU, Non-intensive care unit. ICU, Intensive care unit.

a Pearson’s chi-square test.

b The independent samples T test.

c Mann-Whitney U test.

**Table 3:** The predictive value of SII, NLR, MLR and PLR on the ICU admission.

Variables	AUC	95%CI	Cutoff	Sensitivity	Specificity	*p* value
SII	0.654	0.535–0.774	1145.175	55.60%	74.50%	0.011
NLR	0.701	0.588–0.815	3.906	70.40%	71.10%	0.001
MLR	0.608	0.492–0.725	0.205	92.60%	26.80%	0.074
PLR	0.542	0.411–0.673	236.859	29.60%	88.60%	0.487

AUC, Area under the curve; CI, Confidence intervals; SII, Systemic immune-inflammation index; NLR, Neutrophil-to-lymphocyte ratio; MLR, Monocyte-to-lymphocyte ratio; PLR, Platelet-to-lymphocyte ratio; ICU, Intensive care unit.

## 4 Discussion

To our knowledge, this is the first study to explore differences in SII, NLR, MLR, and PLR between AE patients and healthy individuals and compare the predictive value of the NLR, SII, MLR and PLR for ICU admission in AE patients and investigate the relationship between these four inflammatory biomarkers and the severity of AE, which was assessed by the CASE scale and mRS scale at the same time. In our study, AE patients had higher SII, NLR, MLR and PLR than HCs. Our results showed that the SII and NLR strongly related to the severity of AE. Furthermore, by analyzing ROC curves, we found that the NLR appeared to be the best predictor of ICU admission for AE patients, while PLR was the weakest predictor.

AE is a kind of highly disabling neurologic disorder characterized by brain inflammation and neural circuit damage (1). Both innate immunity and adaptive immunity are involved in the occurrence and development of AE (12). Many immune cells participate in this process, in which they act as important effectors and regulators and activate the downstream inflammatory cascade (13). Consistent with our results, we found that neutrophils, lymphocytes and monocytes are significantly higher in AE patients than in healthy controls. Neutrophils, lymphocytes and monocytes are representative cells of innate immunity and play an important role in the inflammatory response in AE. In the physiological conditions of AE, large amounts of cytokines, such as interleukin 1 beta (IL-1β), IL-6, and tumor necrosis factor-alpha (TNF-*α*), are released by neutrophils involved in the immune cascade and recruited monocytes to the inflammatory sites. Activated monocytes contribute to further disruption and increased permeability of the BBB. Meanwhile, activated monocytes also participate in the activation of T-lymphocytes and B-lymphocytes, thereby initiating adaptive immunity. Under the influence of CD4+ T cells, B cells differentiate into plasma cells, which subsequently produce autoimmune antibodies that contribute to the pathogenesis of AE (14, 15). Studies have shown that neuroinflammation can be effectively inhibited by blocking lymphocyte migration into the central nervous system (16). In addition, platelets can also activate monocytes and dendritic cells through CD40-CD40L interaction, promoting antigen-presenting cells to transmit antigen information to T cells to boost adaptive immunity (17).

Originating from blood routine, NLR, SII, MLR and PLR are convenient and inexpensive markers to assess the state of the systematic inflammation. Consistent with our study, some studies found that NLR was significantly higher in AE patients than healthy controls (18, 19). Additionally, those studies revealed that severe patients tended to have high NLR levels, which was an independent risk factor for severe of AE. However, the number of patients included in this study was small. The use of the mRS scale alone, as well as reliance on a single indicator such as NLR, limited the value of their results. Another study has evaluated the correlation between the SII and other related inflammatory markers with treatment response at 30 days in AE patients. After correcting for confounding factors, the multivariable logistic analysis found that high SII was associated with poor response to first-line immunotherapy at 30 days, but not with PLR and NLR. The study excluded all patients on second-line therapy, which may introduce selection bias. Therefore, their findings have limited reference value for clinicians in optimizing clinical treatment decisions (20). Several studies have examined the correlation between the severity of AE with NLR and MLR, and their potential predictive value for a one-year prognosis. (21–23). However, the conclusions lacked persuasiveness. On the one hand, these inflammatory biomarkers were changeable, so the tests at admission cannot reflect the long-term state of the body. On the other hand, the follow-up at 1 year was taken by telephone, which was lack of accuracy. In addition, the mRS was used as the standard of evaluation in the above studies, which has great limitations when assessing non-motor symptoms in AE patients.

In our study, patients in the ICU group showed significantly higher SII and NLR than the non-ICU group, but MLR and PLR were similar in the two groups. According to ROC analysis, the NLR displayed the best prediction of ICU admission among those four biomarkers, suggesting that the NLR is a more effective and early indicator of ICU admission in AE patients. We further analyzed the correlation of those four biomarkers with the severity of AE, which was assessed by the CASE scale and mRS scale, respectively. Our data showed that SII and NLR were correlated with the CASE score and mRS score, but MLR and PLR had a positive correlation only with the CASE score. The mRS scale is designed to evaluate the motor function and the ability to walk after a stroke, and now it is used to measure disability of other diseases, too (24). AE patients have a variety of non-motor symptoms including seizures, abnormal behavior, memory deficit, speech dysfunction and autonomic instability (1). The CASE scale has been proven to be convergent in validity with mRS and capable of distinguishing between patients with similar mRS scores in many studies since 2019 (25, 26). In our study, the correlation coefficients of SII, NLR, MLR and PLR with the CASE score were greater than those with the mRS score. Our findings indicated that the CASE scale is more sensitive than the mRS scale when it comes to assessing the severity of AE. Furthermore, SII and NLR performed better than MLR and PLR in identifying potentially severe cases early, which helps clinicians optimize treatment strategies for AE patients. Additionally, patients with a high NLR should be given increased attention and undergo early intervention to prevent deterioration of their condition.

There are several limitations to this study. First, our study has a retrospective design. Second, we did not analyze the subtypes of AE or the correlation of AE antibody titers in serum and CSF, so the results may not be extrapolated to all patients with AE. Additionally, we only recorded inflammatory indexes within 24 h after admission. However, the inflammatory response may evolve over the first few days following AE onset. Thus, prospective, large-sample, multicenter studies should be conducted to explore the changes and predictive value of these inflammatory biomarkers at different time points, which may be associated with disease progression, long-term prognosis, and strategies for immunotherapy.

In conclusion, the NLR, SII, MLR and PLR at admission significantly increased in AE patients and were positively correlated with the severity of AE. Among these biomarkers, the NLR has the best performance in predicting ICU admission of AE patients. The NLR is a more effective indicator, which could serve as a practical and reliable biomarker in monitoring disease progression and identifying potentially severe patients. In addition, we recommend the priority of the CASE scale in assessing the severity of AE in clinical practice.

## Data Availability

The raw data supporting the conclusions of this article will be made available by the authors, without undue reservation.
